# Dog-walking behaviours affect gastrointestinal parasitism in park-attending dogs

**DOI:** 10.1186/1756-3305-7-429

**Published:** 2014-09-04

**Authors:** Anya F Smith, Christina AD Semeniuk, Susan J Kutz, Alessandro Massolo

**Affiliations:** Department of Ecosystem and Public Health, Faculty of Veterinary Medicine, University of Calgary, 3280 Hospital Drive NW, Calgary, AB T2N 4Z6 Canada; Great Lakes Institute for Environmental Research, University of Windsor, 401 Sunset Ave, Windsor, ON N9B 3P4 Canada

**Keywords:** Dog, Gastrointestinal parasite, Zoonotic, Parasitism, Urban parks, Epidemiology

## Abstract

**Background:**

In urban parks, dogs, wildlife and humans can be sympatric, introducing the potential for inter- and intra-specific transmission of pathogens among hosts. This study was conducted to determine the prevalence of zoonotic and non-zoonotic gastrointestinal parasites in dogs in Calgary city parks, and assess if dog-walking behaviour, park management, history of veterinary care, and dog demographics were associated with parasitism in dogs

**Methods:**

From June to September 2010, 645 questionnaires were administered to dog owners in nine city parks to determine behavioural and demographic factors, and corresponding feces from 355 dogs were collected. Dog feces were analyzed for helminth and some protozoan species using a modified sugar flotation technique and microscopic examination, a subsample was analyzed for *Giardia* spp. and *Cryptosporidium* spp. using a direct immunofluorescence assay. Descriptive and multivariate statistics were conducted to determine associations among behaviours, demographics, and parasite prevalence and infection intensities

**Results:**

Parasite prevalence was 50.2%. *Giardia* spp. (24.7%), *Cryptosporidium* spp. (14.7%), and *Cystoisospora* spp. (16.8%) were the most prevalent parasites. Helminth prevalence was low (4.1%). Presence of *Giardia* spp. was more likely in intact and young dogs; and infection with any parasite and *Giardia* spp. intensity were both positively associated with dogs visiting multiple parks coupled with a high frequency of park use and off-leash activity, and with being intact and young. *Cryptosporidium* spp. intensity was associated with being intact and young, and having visited the veterinarian within the previous year

**Conclusions:**

Our results indicate a higher overall prevalence of protozoa in dogs than previously found in Calgary. The zoonotic potential of some parasites found in park-attending dogs may be of interest for public health. These results are relevant for informing park managers, the public health sector, and veterinarians.

## Background

Urban parks are a common destination for owners and their dogs [1]. Parks encourage a broad scope of healthy physical activity, including dog-walking [2], and are areas of socialization for dogs and their owners [3, 4]. However, urban parks are also often confined areas where wildlife, dogs, and humans are sympatric, introducing the potential for disease transmission among domestic and wild animals. Environmental contamination and undisposed dog feces positive for zoonotic and non-zoonotic gastrointestinal (GI) parasites have been reported in urban parks [5–7], suggesting parks as potential sources of GI infection for dogs, humans, and wildlife.

Numerous studies investigating the epidemiology of GI parasites circulating in dogs have been conducted in urban areas worldwide. Demographics, geographic location, seasonal trends, and husbandry, have all been considered as risk factors for parasitism [5, 8–10]. Potential risks for parasitism in dogs associated with park use have been investigated as well, although incomprehensively within multi-factor studies [5, 8]. Only one study has focused solely on the direct association between park use and parasitism in dogs, and found a significant association between park attendance and *Giardia* spp. and *Cryptosporidium* spp. infections [11]. In Canada, few studies investigating GI infections in urban dogs have been carried out, and even fewer in Western Canada [5, 12–14], despite that an estimated 33% of Canadians own dogs [15].

In Calgary, over 10,000 hectares of parkland are available for use by people and their dogs [16]. The potential for terrestrial and water contamination from wild and domestic canid feces, and from infected intermediate and paratenic rodent hosts in these parks introduces the possibility of infection in dogs. The potentially zoonotic parasites *Echinococcus multilocularis*, *Toxocara canis*, *Ancylostoma caninum, Giardia* spp., and non-zoonotic *Toxascaris leonina* and *Uncinaria stenocephala,* have been found in coyotes (*Canis latrans*) in Calgary [17–19]. With the exception of Taeniidae, these parasites have also been found in some shelter and homed dogs in Calgary [13], although potential associations to park use were not investigated. The occurrence of these parasites in homed dogs in Calgary [13] and the potential for increased exposure of park-attending dogs to sources of infection [11], prompted our assessment of enteric parasites in Calgary park-attending dogs.

This study focused on a subpopulation of urban dogs walked in city parks. In particular, we aimed to A) provide an inventory of enteric parasites in park-attending dogs in Calgary, and B) identify risk factors for dog parasitism associated with dog-walking behaviour in city parks, form of park management (on-leash, off-leash, or mixed off/on-leash), history of veterinary care, and dog demographics. We hypothesized urban park-walking to be a key risk factor for GI parasite infections in city dogs. In particular, we predicted the use of off-leash versus on-leash parks, the number of total parks visited, frequency of park use, and off-leash activity level within parks to be positively associated with dog parasitism.

## Methods

### Study design and study areas

We used an observational, cross-sectional study design. The study was conducted in Calgary, Alberta, Canada (51°50 N, 114°55’W). Nine city parks were selected on the basis of park management and included three off-leash, three on-leash, and three mixed management parks (parks with both on-leash and off-leash areas). Off-leash parks included Southland (SL), Nose Creek (NC) and Edworthy (EDW); on-leash parks were Stanley (SP), Fish Creek Provincial (FCPP), and Weaselhead (WSH); and mixed management sites were River (RP), Bowmont (BOW), and Nosehill (NH) Parks. All parks were used by coyotes.

### Interview protocol and questionnaire design

Each park was visited once per week from June to September 2010. Dog owners were approached opportunistically in the park and asked to participate in a questionnaire and to provide their dogs’ feces. A total of 635 surveys were conducted; an average of 71 (±4.1) per park.

The questionnaire consisted of 23 questions arranged within three sections: screening, dog demographics and human behaviour, and personal information. Dog demographic variables included breed, gender, age class, and spay or neuter status. Human behaviour variables included 1. Dog-walking behaviours: level of off-leash activity, park visitation frequency, whether the owner and dog visited one or greater than one park on a regular basis (park focus), and the number parks visited in addition to the most frequented park (number of additional parks; NAP); 2. Veterinary care history (whether or not the dog had visited the veterinarian in the previous year). Questions were open or close-ended. Off-leash activity was ranked on a four-point scale and ranged from “never” to “always”. Visitation frequency was ranked on a six-point scale and ranged from “rarely” to “everyday” (Table 1). Research involving human subjects was approved by the University of Calgary Conjoint Faculties Research Ethics Board (file #: 6498).

**Table 1:** **Survey design including questions, answer options, and scoring**

Section	Question	Answer
1. Screening		
	Are you over the age of 18?	No (0)/Yes (1)
	Is this your dog?	No (0)/Yes (1)
	Does your dog normally defecate in this park?	No (0)/Yes (1)
2. Dog demographics and human behaviour		
*Dog demographics*	What is the age of your dog?	Open
	What is your dog’s breed?	Mixed (1)/Purebred (2)
	What is the gender of your dog?	Male (1)/Female (2)
	Is your dog neutered or spayed?	No (0)/Yes (1)
*Veterinary care history*	Has your dog visited a veterinarian within the last year?	No (0)/Yes (1)/Unknown (2)
	Have you de-wormed your dog in the last 12 months (including heartworm medication)?	No (0)/Yes (1)/Unknown (2)
*Walking behaviour*	How often do you come to this park?	0 Rarely 0–3 Times/yr
		1 Occasionally <1/mnth
		2 Infrequently 1–3 days/mnth
		3 Regularly 1/week
		4 Often 2–6 days/week
		5 Everyday 1/day
	When do you come to this park?	Weekdays (1)/weekends (2)/both (3)
	If a mixed park: management areas used	Off-leash (0)/On-leash (1)/both (2)
	How often do you let your dog off-leash in this park?	Never (0)/Rarely (1)/Sometimes (2)/Always (3)
	Do you visit any other parks in Calgary?	No (0)/Yes (1)
*If yes:*	Which parks?	Open
	Which one of these additional parks do you visit most often (P1)?	Open
	How often do you go to this park (P1)?	0 Rarely 0–3 Times/yr
		1 Occasionally <1/mnth
		2 Infrequently 1–3 days/mnth
		3 Regularly 1/week
		4 Often 2–6 days/week
		5 Everyday 1/day
	When do you go to this park?	Weekdays (1)/weekends (2)/both (3)
	Park management type?	Off-leash (0)/On-leash (1)/both (2)/unknown (3)
	How often do you let your dog off-leash in this park?	Never (0)/Rarely (1)/Sometimes (2)/Always (3)
3. Personal information		
	What is your name?	Open
	What is your postal code?	Open
	What is your email address?	Open

### Fecal sampling protocol

Dog feces corresponding to questionnaires were opportunistically collected from owners at the end of their walk, or by requesting owners to collect a sample in a provided, labeled bag during their walk and deposit it into one of the coolers set up at select park exit points. A total of 550 samples corresponding to questionnaires were collected. The samples were kept at -80°C for 72 hours to deactivate *Echinococcus* spp. eggs [20]. Samples were then transferred to -20°C until laboratory analysis. The time interval between beginning of sample collection and laboratory analysis was approximately six months.

A subsample of 355 of the 550 samples collected was selected for laboratory analysis to achieve an average sample size of 39.4 (±0.47) samples per park and to deliberately maximize the number of dogs and owners that frequently attended parks, so as to determine the risk factors for parasitism specifically relevant to park attendees. Selection of cases for laboratory analysis were prioritized hierarchically according to the following criteria: 1) dogs with owners who showed a higher fidelity to one of the study sites than to parks not included in the study; 2) those with a single park focus; 3) those with a high visitation frequency to their most frequented park (MFP); 4) dogs 12 months of age or younger to maximize the juvenile contingent (Table 1). Selection using these criteria was made to ensure even sampling across parks, as well as including only one dog per household (i.e., if multiple dogs per household; the dog fulfilling the greatest number of criteria was chosen). Focusing on dog owners with an MFP equal to one of the nine study sites and who had a single park focus maximized representation of behavioural, demographic, and parasitic status across sites. Samples were prioritized to fulfill the highest number of selection criteria but it was not possible for all samples to fulfill all of the criteria. Once MFP and park focus were prioritized for those with an MFP equal to one of our nine study sites and a single park focus, and stratified into both prioritized and opposing groups, remaining samples were hierarchically selected in order of the highest to lowest ranks of park visitation frequency and, finally, selected for juvenile dogs.

### Laboratory analysis

Two sections of 2 grams of feces were removed from each fecal sample and analyzed separately for helminth eggs and coccidian oocysts using a modified Wisconsin double centrifugation technique [21] and subsequent microscope examination (Leika DME, 10X-40X). Intensity of eggs or oocysts per gram (epg or opg) was recorded and averaged between duplicate samples. Due to funding and time constraints, a subsample of 253 was selected from the 355 samples and investigated for *Giardia* spp. cysts and *Cryptosporidium* spp. oocysts. The subsample was selected to maximize equal representation of dogs across parks with an average of 28 per park (±0.20). Two grams of feces were concentrated and analyzed using a direct immunofluorescence assay (DFA) and a Fluorescein-labeled dual monoclonal antibody reagent (Waterborne Inc., New Orleans, Louisiana, USA). Cysts per gram (cpg) and opg were recorded.

Polymerase Chain Reaction (PCR) was performed following a described methodology of Multiplex-PCR for specific diagnosis of *E. multilocularis*, *E. granulosus*, and *Taenia* spp. [22]. Taenidae eggs collected from the fecal float of one dog sample (53 epg) were lysed in 50 μl of DNA extraction buffer [500 mM KCl, 100 mM Tris–HCl (pH 8.3), 15 mM MgCl_2_, 10 mM DTT, and 4.5% Tween 20] containing 4 μl of Proteinase K. Two μl of diluted lysate (1:20 in dH2O) were used to run the multiplex-PCR. PCR products were electrophoresed on 2% agarose gel and visualized with UV light.

### Data analysis

Dog owners using one of our nine study sites as their MFP were selected for descriptive, Chi-Square, and non-parametric analyses to ensure representation of dogs attending our study sites (sample n = *345*; subsample n = *251*). Estimated true parasite prevalence was calculated by incorporating parameters of imperfect screening tests [23] and median intensity were calculated overall, and stratified for dog age (≤or > 12 months old, juvenile and adult classes, respectively). Confidence intervals were also calculated using the Sterne estimator [24] and upper and lower limits calculated for intensity values. Median intensity values were rounded up to the nearest whole number. Helminth species were grouped together due to the low prevalence of individual species. Mean age of juvenile dogs was calculated to reflect the proportion of puppies (≤6 months old) versus dogs older than six months comprising the juvenile age category. Chi-square exact test [25] was used to determine differences in occurrence of *Giardia* spp., *Cryptosporidium* spp., *Cystoisospora* spp., and helminths overall, within each age class and between age classes. Differences among median intensities of *Giardia* spp, *Cryptosporidium* spp., and *Cystoisospora* spp. between age classes were compared using Mann–Whitney Test for two independent samples [25].

To analyze the association between dog-walking behaviours or dog demographics and parasitism, we used logistic regression to predict presence or absence of different parasites, and linear regression to model infection intensity. Scores for off-leash activity and park visitation frequency in the MFP and second most frequented park (P1) were both pooled into two classes and summed (MFP + P1 values for off-leash activity or visitation frequency) to produce totals reflective of overall off-leash activity and visitation frequency. To accurately make this calculation, only dogs with owners whose MFP was the park where the questionnaire was conducted were included in analysis. MFP low frequency ranks of 0 and 1 (i.e., low fidelity to the selected parks) were filtered out to target park-attending dogs and to ensure the representation of the MFP (Table 1).

Principal Components Analysis for Categorical Data (CATPCA) [26] was performed to reduce the number of independent variables (behaviours, dog demographics and veterinary care history) and to control for correlated variables in multivariate analyses. CATPCA generates an additive model with as many dimensions as there are groups of tightly associated variables in the data [26]. Each dimension is an equation in which each variable has a coefficient (component loading) that represents its association with that dimension. Variables highly associated with each other have high loading magnitudes in the same component. The intention of the CATPCA was to summarize the high number of dog demographic and dog-walking behaviour variables; therefore, the form of park management was not included in the CATPCA. Two CATPCA dimensions were used in the multivariate models. The variables that did not enter into the CATPCA were entered into the multivariate models independently.

Binary logistic regression [27] was used to quantify relationships between the presence of *any* parasite (helminths or protozoa), of *Giardia* spp., of *Cryptosporidium* spp., or of *Cystoisospora* spp. using the CATPCA dimensions and the variables excluded from the CATPCA (n = *248*). Presence of individual helminth species or genera and *Sarcocystis* spp. were not used as dependent variables due to their low prevalence values.

The relationship between parasite intensities (*Cystoisospora* spp., *Giardia* spp., and *Cryptosporidium* spp.), and the CATPCAs and variables were determined using negative binomial regression [27]. Only positive samples were used in the analysis, i.e. those with intensity values greater than zero (*Cystoisospora* spp. n = *56*; *Giardia* spp. n = *61*; *Cryptosporidium* spp. n = *36*).

Statistical analyses were conducted using SPSS version 20.0 (SPSS, Chicago, Illinois, USA).

## Results

The overall prevalence of dogs infected with at least one parasite was 50.2%. Juveniles comprised 15.9% of the sample. Prevalence of infection in this group was 70%. Adult dogs comprised 84.1% of the sample and prevalence of infection was 46.4% (Table 2). The mean age of juveniles included in the sample (in months) was 9.34 (±0.37; *n* = 55), and 9.30 (±0.41; *n* = 40) in the subsample.

**Table 2:** **Prevalence (%) and median intensity (n/g) of GI parasites in dogs overall and stratified by age class**

	***Giardia***spp.		***Cryptosporidium***spp.		***Cystoisospora***spp.		Helminths	Overall
Age	n/gr ^a^	%	n/gr	%	n/gr	%	%	%
Juv	19.0^b^	55.0	1.0	15.0	32.5	18.2	3.6	70.0
	(1–600)^c^	(38.8-70.2)^d^	(1–200)	(6.7-29.8)	(1–250)	(9.7-30.8)	(.1-11.7)	(53.8-82.9)
		(n = *40*)		(n = *40*)		(n = *55*)	(n = *55*)	(n = *40*)
Adults	2.5	19.0	1.0	14.7	38.0	16.6	4.1	46.4
	(1–500)	(14.2-24.8)	(1–10)	(10.4-20.1)	(1–250)	(12.7-21.2)	(2.0-7.1)	(39.5-53.3)
		(n = *211*)		(n = *211*)		(n = *290*)	(n = *290*)	(n = *211*)
Overall	6.0	24.7	1.0	14.7	38.0	16.8	4.1	50.2
	(1–600)	(19.7-30.5)	(1–200)	(10.7-19.7)	(1–250)	(13.2-21.1)	(2.4-6.7)	(44.0-56.4)
		(n = *251*)		(n = *251*)		(n = *345*)	(n = *345*)	(n = *251*)

^a^Number of cpg, opg, or epg (n/g).

^b^Median intensity values rounded up to the nearest whole number.

^c^Infection intensity intervals (upper and lower limits of epg, opg, or cpg).

^d^95% confidence intervals.

*Giardia* spp. (24.7%), *Cryptosporidium* spp. (14.7%), and *Cystoisospora* spp. (16.8%) were the most prevalent parasites (Table 2). Prevalence of detected helminth species or genera was much lower overall (4.1%) and included *Capillaria* spp. (1.4%), *Eucoleus* spp. (0.60%), *Trichuris* spp*.*(1.2%), *Toxascaris leonina* (0.60%), *Toxacara canis* (0.30%), *Taeniidae* species (0.30%), *Diphylobothrium* spp. (0.30%), and *Uncinaria stenocephala* (0.30%). *Sarcocystis* spp. was also detected (0.30%).

PCR analysis resulted in amplicons of expected 117 and 267 bp indicating positive diagnosis of *E. granulosus* and *Taenia* spp., respectively, and the absence of 395 bp amplicons excluded the presence of *E. multilocularis*. We did not sequence the PCR products of *E. granulosus* and therefore could not confirm this diagnosis.

There were some significant differences in prevalence seen among *Giardia* spp., *Cryptosporidium* spp., *Cystoisospora* spp., and helminths overall, within each age class, and between age classes (Table 3). There was no significant difference between adults and juveniles in infection intensities of *Giardia* spp., *Cystoisospora* spp., or *Cryptosporidium* spp.

**Table 3:** **Significant differences in parasite prevalence within each age class, between age classes, and overall**

	Juveniles ^a^	Adults	Juveniles > adults	Overall
***Giardia***vs. Helminths				
Prevalence (%)	55 > 3.6^b^	19 > 4.1	-	24.7 > 4.1
*X* ^2^	301.2	81.7	-	209.5
df	1	1	-	1
*P*	<0.001	<0.001	-	<0.001
***Giardia*** **vs.** ***Cryptosporidium*** **spp.**		-		
Prevalence (%)	55 > 15	-	-	24.7 > 14.7
*X* ^2^	50.2	-	-	19.8
df	1	-	-	1
*P*	<0.001	-	-	<0.001
***Giardia*** **vs.** ***Cystoisospora*** **spp.**		-		
Prevalence (%)	55 > 18.2	-	-	24.7 > 16.8
*X* ^2^	36.5	-	-	11.5
df	1	-	-	1
*P*	<0.001	-	-	=0.001
***Cryptosporidium*** **spp. vs. Helminths**				
Prevalence (%)	15 > 3.6	14.7 > 4.1	-	14.7 > 4.1
*X* ^2^	14.7	21	-	51.6
df	1	1	-	1
*P*	=0.003	<0.001	-	<0.001
***Cystoisospora*** **spp. vs. Helminths**				
Prevalence (%)	18.2 > 3.6	16.6 > 4.1	-	16.8 > 4.1
*X* ^2^	33.2	76.6	-	104
df	1	1	-	1
*P*	<0.001	=0.004	-	<0.001
**Overall parasitism**				
Prevalence (%)	-	-	70 > 46.4	-
*X* ^2^	-	-	8.9	-
df	-	-	1	-
*P*	-	-	<0.001	-

^a^Age strata. Significant differences in parasite prevalence within juvenile and adult age classes, between age classes (where parasite prevalence is higher in juveniles than adults), and overall.

^b^Example of coding for significant parasite prevalence differences. In this case, prevalence of *Giardia* spp. (55%) is greater than helminths (3.6%) in the juvenile age class.

The final CATPCA analysis included off-leash activity total, park visitation frequency total, park focus, NAP, spay or neuter status, and age and resulted in two dimensions with Eigenvalues of >1, accounting for 78.1% of the total variance (Table 4). Breed, gender, and whether or not the dog had visited a veterinarian in the previous year, as well as park management type did not enter into the CATPCA. The first dimension (exposure) explained 54.5% of the total variance and grouped variables related to off-leash activity total, NAP, park focus, and park visitation frequency total. All variables in this dimension had large positive coefficients (>0.6) with the exception of park focus, which was negatively associated with this dimension. The second dimension (dog demographics) explained 25.6% of the total variance and grouped dog demographic variables such as spay or neuter status and age that had positive high values (>0.7) (Table 4).

**Table 4:** **Results of CATPCA**

	Component loadings	
	Dimension	
Variable	Exposure	Dog demographics
	(1)	(2)
Off-leash activity total	0.911	-
Park visitation frequency total	1.029	-
Park focus	-1.049	-
Number of additional parks visited	0.640	-
Spay/neuter status	0.142	0.919
Age ≤ or > 12 months	-	0.856

Component loading values represent a variable’s association with each dimension. Variables highly associated with each other have high loading magnitudes in the same dimension.

The logistic models highlighted a statistically significant positive association between the presence of *Giardia* spp*.* and the dog demographics dimension (i.e. being juvenile and intact) (Table 5). Overall parasitism was significantly positively associated with exposure (off-leash activity and park visitation frequency totals, park focus, NAP) and dog demographics dimensions (Table 5).

**Table 5:** **Significant results of two multivariate regression models: 1) A binary logistic regression model of the associations between GI parasite presence and dog demographic and exposure dimensions and age, breed, gender, and park management variables; 2) A negative binomial regression model of the associations between GI parasite median intensity and dog demographic and exposure dimensions and age, breed, gender, and park management variables**

Parameter							
Estimates:	Factor	Wald Chi-Square	B	df	***P***value	***LL***	LL ratio ***X*** ^2^
**1)** ***Presence/absence*** ^**a**^ **:**							
*Giardia* spp. (n = *248*)	Dog Demographics Dimension	15.2	-0.499^b^	1	<0.001		
Overall model				7	0.001	-86.9	24.4
Any parasite (n = *248*)	Exposure Dimension	4.9	0.287	1	0.026		
	Dog Demographics Dimension	8.3	-0.366	1	0.004		
Overall model				7	0.006	-111.7	19.7
**2)** ***Infection intensity:***							
*Giardia* spp. (n = *61*)	Exposure Dimension	4.5	0.357^c^	1	0.035		
	Dog Demographics						
	Dimension	7.0	-0.278	1	0.008		
	Female Gender	4.8	0.698	1	0.028		
	Purebred	3.9	-0.620	1	0.048		
Overall model				7	0.001	-318.1	27.1
*Cryptosporidium* spp. (n = *36*)	Dog Demographics Dimension	11.4	-0.751	1	0.001		
	Visited Veterinarian within previous 12 months	8.2	2.894	1	0.004		
Overall model				7	0.001	-87.9	48.8

^a^The reference category for the dependant variable is 0 where 0 = negative and 1 = positive for parasites.

^b^B value magnitude and direction is the slope of the line and indicates the chance that a given dog has a value of 1 relative to the component loadings or independent variables.

^c^B value magnitude and direction is the slope of the line and indicates the direction of infection intensity of a given dog relative to the component loadings or independent variables.

Intensity of *Giardia* spp. infection was positively associated with exposure and dog demographics dimensions, as well as to additional demographics not incorporated in the CATPCA including female gender and mixed breed (Table 5). *Cryptosporidium* spp. intensity was significantly positively associated to the dog demographics dimension, as well as having visited the veterinarian in the previous year (Table 5).

## Discussion

Our results indicated a higher prevalence of protozoa relative to helminths, and *Giardia* spp., *Cystoisospora* spp., and *Cryptospordium* spp. were the most prevalent parasites overall. Adults were parasitized significantly less often than juveniles, findings consistent with other studies [8, 9].

The dominance of protozoa versus helminth infection occurred overall and within both age classes. Higher infection rate with protozoa relative to helminths has also been detected in other parts of the developed world [8]. This may be partially attributed to differences in abundance in the environment, degree of lifecycle complexity, and disease ecology among some protozoa and helminth species [28, 29]. Veterinarians also typically treat for helminths more often than protozoa [30], another likely driver of this result.

Although the prevalence of helminths is clearly lower than protozoa in Calgary park-attending dogs overall, helminth prevalence is likely underestimated. The sensitivity of detecting certain helminth species using sucrose flotation is low [19], thus our use of sucrose flotation likely underestimated the true frequency of helminth infection in dogs**.** Other potential reasons for helminth prevalence underestimates include inability to detect prepatent infections, cyclical shedding of eggs [31–33], and hypobiosis [34]. This finding is particularly important in considering our potential detection of *E. granulosus* in a Calgary dog, and the recent discovery of *E. multilocularis* in coyotes [17–19], and *T. canis* in dogs [13] in the Calgary region. These parasites are zoonotic and the causative agents of potentially serious echinococcosis [35] and toxocariasis [36] in humans, respectively.

We detected a higher prevalence of *Giardia* spp. and *Cryptosporidium* spp. than recently found in dogs in Calgary [13]. Differences in the sensitivities of laboratory techniques and in sampled populations are likely the reasons for the disparity. Joffe *et. al.* [13] investigated parasitism in a study population consisting primarily of homed dogs unscreened for park attendance, and accessed samples through veterinary clinics. We focused on a subpopulation of homed, park-attending dogs; dogs potentially more exposed to sources of infection than non-park-attending dogs, and therefore more likely parasitized. Furthermore, the more sensitive DFA technique used here may partially account for the higher prevalence of *Giardia* spp. and *Cryptosporidium* spp. than zinc sulphate flotation used in the previous study [37]. However, our results also showed a higher prevalence of these two parasites in park-attending dogs than found by Wang *et al.* [11], who compared *Giardia* spp. and *Cryptosporidium* spp. prevalence in Colorado dogs that attended parks, versus dogs that did not attend parks, using an immunofluorescence technique similar to the one used in this study.

*Giardia* spp. was the most prevalent parasite overall and within both age classes. This is not surprising given it is one of the most common GI parasites infecting humans and domestic animals worldwide [38]. *Giardia duodenalis* is the only known species to infect both humans and dogs, but the degree of zoonotic risk is debated [39]. Currently, there are six species of *Giardia* and eight assemblages within the *G. duodenalis* species complex [40, 41]. Several studies in North America confirmed assemblages C and D as the most common strains found to infect dogs, appearing largely host-specific, and non-zoonotic [11, 38]. In addition to host-specific assemblages C and D, assemblages with a broader host range such as A and B have also been found in dogs, although are more commonly seen in humans [42]. However, assemblage-specific infection in dogs can vary geographically, and potentially zoonotic assemblages A and B can even dominate as strains present in dogs in some regions of North America and Europe [43, 44]. Coyotes have been reported to harbour assemblages A, D (Smith *et al.,* unpublished data, [45]), and C in Alberta, and indicated as a possible reservoir for human infection in the province [45]. Direct transmission occurs among humans and animals via contaminated water, food, or physical contact [46]. Host species infected with *Giardia* spp. can be asymptomatic, or present clinical signs ranging from mild to severe diarrhea from malabsorption [47]. *Giardia* spp. is environmentally pervasive; the infective cysts are robust and can survive for long periods in cool, damp soil and water [40], both common park elements and potential sources of infection for park attending dogs. *Cryptosporidium* spp. and *Cystoisospora* spp. were also common protozoa found to infect park-attending dogs. Both parasites are directly transmitted and have a clinical presentation similar to *Giardia* spp. [46–48]. Like *Giardia* spp., *Cryptosporidium* spp. is a very common GI infectious agent worldwide [49], potentially zoonotic, with a robust infectious oocyst [50]. The *Cryptosporidium* genus is currently comprised of 19 species and over 40 genotypes. *Cryptosporidium canis* is the predominant species found in dogs [43, 51] and has also been found in coyotes in Alberta (Smith *et al.,* unpublished data, [45]). Although *C. canis* is potentially zoonotic, it is considered very low risk to humans [51, 52]. Human infections with *Cryptosporidium* are most often caused by host-specific *Cryptosporidium hominis* and less specific *Cryptosporidium parvum* [51]. *C. parvum* has infrequently been reported in dogs [51], but no evidence of infection in dogs with *C. hominis* has been detected in the literature. The appearance of *Cryptosporidium* spp. is an important finding; only two other recent Canadian GI parasite prevalence studies have detected this parasite in dogs, both reporting a lower prevalence than found here [53, 54]. *Cystoisospora* spp. is non-zoonotic, but in extreme cases, can cause hemorrhagic gastroenteritis in dogs [48]. Although moderately prevalent, the overall median intensity was only 38 oocysts per gram. Clinical treatment is recommended for infection intensities at or above 1000 oocysts per gram [48], so concern for the impact of *Cystoisospora* spp. on park-attending dog health in Calgary appears minimal.

Our main hypothesis concerning intensity of park use and parasitism in dogs was supported: level of off-leash activity, number of parks visited, and frequency of park use, were shown to be positively associated to infection with at least one GI parasite species or genera. Behavioural risk factors for general parasitism were found to include [NAP], off-leash activity total, visiting more than one park, and frequency of park visitation. Dogs ranking high in these categories were more likely exposed to potential sources of infection including intermediate hosts, dog and coyote feces, and associated environmental contamination. The lack of detectable association between infection with *Giardia* spp., *Cryptosporidium* spp., or *Cystoisospora* spp. and park management and dog-walking behaviour may indicate a high level of occurrence of these parasites in all parks. With readily transmissible and environmentally resilient (oo)cysts, and as ubiquitous GI parasites found in dogs in general [40, 52], a uniform level of exposure of dogs to these pathogens may exist across parks independent of park management or intensity of park use.

*Giardia* spp. was most likely to infect intact, juvenile dogs, findings also documented in past studies [8]. *Giardia* spp. and *Cryptosporidium* spp. infection intensities were also positively associated with being juvenile and intact. *Giardia* spp. intensity also showed a positive association with off-leash activity, visitation frequency, and number of parks visited, suggesting repeated exposure might contribute to elevated *Giardia* spp. cyst intensities in dogs. Further, *Cryptosporidium* spp. infection intensity was positively associated to visiting the veterinarian within the previous 12 months. Although our study did not explore whether or not infected dogs showed clinical signs, this result suggests that heavily infected dogs may have been symptomatic and subsequently visited the veterinarian.

One limitation of this study to note includes our subsample selection process. We used several criteria principally designed to include those participants who primarily attended one of our study sites and to maximize the number of frequent park users with high park fidelity who owned juvenile dogs, and therefore did not select park-attending dog owners and dogs at random. Although not all samples fulfilled all criteria, this selection process introduces selection bias and may inhibit internal validity to park-attending dogs. However, our intention was not to represent all park-attending dogs, but to determine demographic and behavioural risk factors for those park-attending dogs at a potentially higher risk for parasitism. We selected for juveniles in order to represent the few young dogs that attended parks and stratified for age accordingly. We aimed to represent these individuals across multiple park locales and forms of bylaw and provincial management, all in order to inform the management of high-risk behaviours. Also, we endeavoured to avoid respondent fatigue by surveying dog owners about select behaviours posing potential risks for parasitism in dogs, and did not attempt exhaustive data collection on this topic. We acknowledge other potential risk factors such as total number and species of animals in the home, tendency to drink from still water sources in parks etc. Lastly, we were not able to conduct an a priori power calculation due to the lack of published information on park-attending dog population size and estimated prevalence. Our prevalence estimates incorporated all available alternative data to improve estimated true prevalence such as estimates of sensitivity and specificity of our diagnostic tests, when known.

The outcomes of our study provide information for further epidemiological investigation and disease control interventions, and are beneficial to veterinarians for diagnostic purposes, park and wildlife managers, the public health sector, and dog owners. The positive associations found between intensity of park use and general parasitism, and *Giardia* spp. infection intensity, suggests that park attendance may pose a risk for parasitism in dogs. Prioritizing the removal of dog litter in parks is recommended to reduce and manage risk. Clearly, reducing the burden of dog feces in parks could have a positive health impact for dogs and humans by reducing infection risk, but cleaner parks may also offer an indirect health benefit by providing greater incentive to engage in physical and social activity. Dog litter left behind can be a deterrent to park use [2], and consequently, the accumulation of dog litter in some Calgary parks (Massolo *et al.*, unpublished data) could discourage dog owners and non-dog owners from engaging in physical and social activity in these areas. Management strategies to reduce infection risk and promote park activity could include education campaigns highlighting the importance of picking up after dogs with respect to human, dog, and wildlife health, and increasing the frequency of dog feces cleanup in parks by city services. Veterinarians could also contribute to managing parasite transmission by performing regular examinations for protozoa in addition to helminths and educating clients about responsible park use.

Future research should focus on determining the public health significance of *Giardia* spp. and *Cryptosporidium* spp. infected dogs by conducting molecular studies to strain type these parasites. Also, clarifying the level of risk park attendance poses for GI parasitism in dogs is recommended by expanding the sample to 1) investigate *Giardia* spp. and *Cryptosporidium* spp. prevalence and strains in coyotes sympatric with park-attending dogs and; 2) include non-park-attending dogs as a reference group.

## Conclusions

The prevalence of *Giardia* spp. and *Cryptosporidium* spp. in park-attending dogs is moderate to high. Certain behaviours within urban parks may contribute to parasitism in dogs, introducing potential implications for dog and human health. Public education and removal of dog feces is recommended in parks to maximize enjoyment and minimize parasite transmission risk.
